# Appendix

**Published:** 1834-06-01

**Authors:** 


### APPENDIX,

OR

### Index to the Series of Four Annual Volumes, from April 1820 to June 1824.

| | Vol. | Page |
|---|---|---|
| **A** | | |
| Abdomen, fungoid tumor in the.... | 1 | 463 |
| Abdomen, gun-shot wounds of, by Baron Larrey .............. | 2 | 260 |
| Abdominal tumor................ | 4 | 220 |
| Abdominal pressure as a means of diagnosis .................. | 4 | 569 |
| Abercrombie, Dr. on apoplexy ..... | 1 | 1 |
| Abercrombie, Dr. on the intestinal canal .................. | 1 | 161 |
| Abercrombie, Dr. on consumptive diseases ................. | 1 | 751 |
| Abernethy on life ................ | 3 | 253 |
| Abortion, a species of ............ | 2 | 416 |
| Abortion, Mr. Ward on........... | 4 | 699 |
| Abscess behind the œsophagus. .... | 2 | 518 |
| Abstinence, remarkable case of..... | 3 | 49 |
| Accretion of heart and pericardium.. | 4 | 548 |
| Acetate of morphia, preparation of.. | 4 | 208 |
| Acid baths, Mr. Coyne on ......... | 3 | 510 |
| Acids, their chemical qualities...... | 2 | 315 |
| Acupuncture, Mr. Churchill on .... | 2 | 431 |
| Acupuncture.................... | 4 | 956, 958 |
| Adams, Sir W. on artificial pupil... | 1 | 71 |
| Adams, Sir William, his claims .... | 2 | 449 |
| Adipocere, Dr. Ure on ............ | 2 | 321 |
| Alcock, Mr. on bronchitis......... | 1 | 325 |
| Alcock, Mr. on congenital division of the palate ............... | 1 | 440 |
| Alcock, Mr. on medical education .. | 4 | 521 |
| Alentejo, med. topography of ...... | 2 | 589 |
| Alibert, M. on tinea capitis ........ | 2 | 534 |
| Alibert, Dr. on prurigo formicans. .. | 3 | 779 |
| Alkalies, their properties .......... | 2 | 316 |
| Allan, Mr. on acute mania......... | 4 | 939 |
| Aloes, various preparations of...... | 1 | 89 |
| Amaurosis....................... | 2 | 378 |
| Amaurosis, Dr. Weller on......... | 2 | 650 |
| Amaurosis, Mr. Stevenson on...... | 2 | 788 |
| Amenorrhœa, Dr. Lavagna on ..... | 4 | 700 |
| America, medical science in........ | 1 | 496 |
| Amesbury, Mr. on fractures of the thigh, &c. .................. | 3 | 915 |
| Ammonia as a counter-irritant. .... | 1 | 456 |
| Amputation, proper period for operating.................. | 2 | 176 |
| Amputation during the progress of gangrene .................. | 2 | 186 |
| Amputation at the hip-joint. ...... | 2 | 194 |
| Amputation without ligature of arteries ...................... | 2 | 248 |
| Amputation at the hip-joint........ | 4 | 947 |
| Amputation, remarks on .......... | 4 | 949 |
| Amputation, tourniquets in........ | 4 | 953 |
| Anasarcous limbs, puncture of..... | 3 | 182 |
| Anatomico-pathological researches by Dr. Jackson.............. | 4 | 487 |
| Anatomy, surgical, Mr. Anderson's.. | 3 | 357 |
| Andalusian fevers, Dr. Jackson on .. | 2 | 485 |
| Anderson, Mr. on surgical anatomy, | 3 | 357 |
| Anderson, Dr. on yellow fever...... | 4 | 1009 |
| Andral on the intestinal canal...... | 4 | 673 |
| Aneurism, cases of............... | 1 | 741 |
| Aneurism of the groin ... ........ | 2 | 269 |
| Aneurism, Sir Astley Cooper's pathology of ................. | 2 | 743 |
| Aneurism, Mr. Coates' case of...... | 2 | 870 |
| Aneurism, Mr. Salmon's case of.... | 2 | 876 |
| Aneurism of heart, curious case of.. | 3 | 20 |
| Aneurism, cases of, by Mr. Todd.... | 4 | 77 |
| Aneurism, mistaken............... | 4 | 919 |
| Aneurism, axillary ............... | 4 | 954, 958 |
| Angina pectoris, causes of......... | 2 | 565 |
| Angina pectoris, Dr. Good on...... | 3 | 809 |
| Animation, suspended, Mr. Whiter on | 1 | 671 |
| Antigua, medical topography of .... | 2 | 592 |
| Antimoniated ointment, Dr. Jenner on | 3 | 89 |
| Antimoniated ointment in agues.... | 4 | 701 |
| Anus, prolapsus, &c. of the........ | 1 | 208 |
| Anus, constriction of.............. | 3 | 328 |
| Aorta, obliterated, case of......... | 1 | 491 |
| Aorta, aneurism of, Mr. Hunter on.. | 4 | 767 |
| Apoplexy, Dr. Cooke on ........ | | |
| Apoplexy, Dr. Abercrombie on. .. | | |
| Apoplexy, M. Bricheteau on ..... | 1 | 1 |
| Apoplexy, M. Rochoux on....... | | |
| Apoplexy, M. Serres on......... | | |
| Apoplexy, Morgagni's remarks on .. | 3 | 548 |
| Apothecaries, associated, letters to.. | 1 | 204 |
| Apothecaries-Surgeon, report of.... | 2 | 454 |
| Apothecaries' transactions......... | 4 | 519, 762 |
| Arachnitis, Duchatelet & Martinet on, | 2 | 695 |
| Argus, No. 1, on medical conduct .. | 2 | 222 |
| Argus, No. 2, obstinate pervigilium, | 2 | 224 |
| Argus, No. 4, Sir Charlatan Glanderman ..................... | 2 | 224 |
| Aristotle on life................. | 3 | 250 |

| | Vol. | Page |
|---|---|---|
| Armstrong, Dr. on fever | 3 | 393 |
| Arnott, Mr. on the urethra | 1 | 727 |
| Arsenic, Dr. Paris' test for | 1 | 89 |
| Arsenic, Dr. Gregory on | 2 | 871 |
| Arsenic, poisoning by | 3 | 192<br>203 |
| Arsenic, poison of | 4 | 221 |
| Arson, Dr. Paris on | 4 | 601 |
| Arteria innominata, ligature of the | 1 | 546 |
| Arteries, inflammation of | 2 | 50 |
| Arteries, wounds of | 2 | 266 |
| Arteries, on the vacuity of | 2 | 443 |
| Arteries, non-pulsation of | 3 | 566 |
| Arteritis, case of | 1 | 486 |
| Arteritis, Mr. Golding's case of | 2 | 439 |
| Arteritis, case of | 2 | 683 |
| Arteritis | 3 | 194 |
| Artery, iliac, ligature of | 1 | 758 |
| Artificial pupil, Guthrie and Adams on | 1 | 71 |
| Ascarides, remedy for | 4 | 206 |
| Ascension, fever at | 4 | 916 |
| Ascites cured by pressure | 3 | 189 |
| Ascites, remarkable case of | 3 | 425 |
| Asthma, Dr. Parry's pathology of | 3 | 20 |
| Asylum, Glasgow Lunatic | 4 | 775 |
| Attraction, Dr. Ure on | 2 | 316 |
| Averill's, Mr. operative surgery | 4 | 945 |
| Axillary aneurism, Mr. Mayo's case, | 3 | 390 |
| Axillary aneurism operated on | 3 | 402 |
| Axillary artery, rupture of | 4 | 951 |

B

| | Vol. | Page |
|---|---|---|
| Bacot, Mr. on syphilis | 2 | 598 |
| Baillie, Dr. on paraplegia | 1 | 375 |
| Baillie, Dr. estimate of his character, | 4 | 734 |
| Ballingall, Dr. on syphilis | 1 | 565 |
| Bampfield, Mr. on variola & varicella | 3 | 198 |
| Bann, contagious fever in H. M. S. | 4 | 916 |
| Barclay, Dr. on life, &c. | 3 | 225 |
| Barclay, Dr. answers to | 4 | 868 |
| Barlow, Mr. on surgery & midwifery, | 3 | 64 |
| Barlow, Mr. on the placenta | 3 | 306 |
| Baron, Dr. on tuberculous diseases | 3 | 785 |
| Bath, mineral waters of | 1 | 598 |
| Bath waters, on | 3 | 188 |
| Bayle, M. on œdema of the glottis | 4 | 452 |
| Bedlam, sketches in | 4 | 511 |
| Beer, M. on artificial pupil | 1 | 75 |
| Bell, Mr. C. on the urinary organs | 1 | 264 |
| Bell, Mr. Charles, on the nerves | 2 | 809 |
| Bell, Mr. on cancer mammæ | 4 | 195 |
| Belladonna, observations on | 3 | 412 |
| Berezina, passage of, by the French, | 2 | 245 |
| Berlinghieri, Prof. on lithotomy | 2 | 337 |
| Bibliographical Record | 2 | 230 |
| Bile, Mr. Brodie on the | 4 | 186 |
| Biliary calculi, Mr. Brayne on | 4 | 791 |
| Bingham, Mr. on strictures of the urethra | 1 | 528 |
| Biography of Dr. J. Gregory | 2 | 216 |
| Biography of Dr. Baillie | 4 | 734 |
| Bistouri cachée proposed by Mr. Barlow | 3 | 70 |
| Bladder, wounds of | 2 | 262 |
| Bladder, Mr. Bingham on the | 3 | 767 |
| Blane, Sir G. on vaccination | 1 | 702 |
| Blane's, Sir Gilbert, dissertations | 3 | 733 |
| Blepharophthalmitis | 2 | 630 |
| Blicke, Dr. on cephalitis | 3 | 178 |
| Blood, transfusion of | 1 | 117 |
| Blood, foreign matters in | 3 | 416 |
| Bloodletting, excessive, effects of | 1 | 201 |
| Bloodletting in hæmorrhagic diseases | 2 | 396 |
| Bloodletting in hydrothorax | 4 | 942 |
| Blue disease, remarks on | 4 | 924 |
| Blumenbach on organization | 3 | 236 |
| Blundell, Dr. on transfusion of blood | 1 | 117 |
| Bony tumor of the skull, case of | 1 | 98 |
| Bourne, Dr. case of cardiac disease | 3 | 571 |
| Boyer, M. on fissures of the anus | 3 | 328 |
| Boyle, Mr. on Indian cholera | 2 | 85 |
| Brain, erethism of, by Dr. Nicholl | 2 | 361<br>527 |
| Brain, masked disease of | 2 | 440 |
| Brain, morbid anatomy of | 2 | 559 |
| Brain, on concussion and compression of | 2 | 740 |
| Brain, remarkable wound of | 3 | 428 |
| Brain, pathology of, in mania | 3 | 434 |
| Brain, M. Lallemand on diseases of, | 3 | 465 |
| Brain, painful affection of | 4 | 463 |
| Brain, destruction of, in a fœtus | 4 | 800 |
| Brayne, Mr. on biliary calculi | 4 | 791 |
| Brera, Prof. on verminous diseases | 1 | 549 |
| Brera, Professor, on iodine | 3 | 757 |
| Breschet, M. on melanose | 3 | 208 |
| Bricheteau, M. on apoplexy | 1 | 1 |
| Bridlington epidemic, account of | 2 | 203 |
| Briggs, Mr. James, on scirrhus and cancer | 4 | 172 |
| Brodie, Mr. on the bile | 4 | 186 |
| Brodie, Mr. on extraction of calculi, | 4 | 804 |
| Bronchitis, Drs. Hastings, Broussais Villermé, Harrison, and Mr. Alcock on | 1 | 325 |
| Bronchocele cured by iodine | 2 | 322 |
| Bronchocele, Mr. Hutchison on | 2 | 866 |
| Bronchocele, case of, operated on | 3 | 194 |
| Bronchocele, Mr. Austen on | 4 | 193 |
| Bronchocele, iodine in | 4 | 801 |
| Bronchotomy | 4 | 213 |
| Bronchotomy, Dr. Burgess on | 4 | 339 |
| Broussais' histoire des phlegmasies | 1 | 161<br>325 |
| Broussais, M. on gastritis and enteritis | 2 | 1 |
| Brucine in paralysis | 4 | 944 |
| Brunonianism in Italy | 2 | 747 |
| Bull, Dr. on cancer of the lip | 4 | 464 |
| Bullen, Mr. case of ligature of the subclavian | 4 | 954 |
| Burgess, Dr. on bronchotomy | 4 | 339 |
| Burnett, Dr. W. on mercurial vapour, | 4 | 1010 |

| | Vol. | Page |
|---|---|---|
| Burns' principles of midwifery | 1 | 579 |
| Burrows, Dr. on insanity | 1 | 237 |
| Buxton, mineral waters of | 1 | 590 |
| Byron, Dr. on coagulum of blood in the bladder | 4 | 316 |
| **C** | | |
| Cabanis' remarks | 3 | 243 |
| Cadiz fevers, Dr. Jackson on | 2 | 485 |
| Cæcum, ulceration of | 4 | 470, 739 |
| Cæcum, scirrhous, mistaken | 4 | 919 |
| Cæsarian operation successful | 3 | 324 |
| Cæsarian operation | 4 | 964 |
| Calculi, urinary, tabular view of | 1 | 93 |
| Calculi, renal, Mr. Earle on | 2 | 436 |
| Calculi, Sir Astley Cooper on extraction of | 2 | 874 |
| Calculi, on extraction of, Sir Astley Cooper | 4 | 803 |
| Calculous diathesis, Dr. W. Philip on | 1 | 607 |
| Calculous disorders, Dr. Prout on | 2 | 89 |
| Calhoun, Dr. on paralysis | 2 | 279 |
| Caloric, Dr. Ure on | 2 | 317 |
| Campaign, Russian, in 1812 | 2 | 237 |
| Campbell, Dr. on uterine hæmorrhage | 1 | 470 |
| Cancer, the present modes of treating | 2 | 733 |
| Cancer of the lip, M. Richerand on | 3 | 427 |
| Cancer of the lip, operation for | 3 | 696 |
| Cancer, Professor Scarpa on | 4 | 172 |
| Cancer, Mr. Bell on | 4 | 195 |
| Cancer, cured by oxymurias hydrag., | 4 | 222 |
| Cancer, chimney-sweepers' | 4 | 799 |
| Cancer of the penis | 4 | 837 |
| Carbuncle, Mr. Swallow on | 4 | 934 |
| Carcinoma uteri, Mr. Clarke on | 3 | 85 |
| Carcinoma mistaken | 3 | 420 |
| Cardiac diseases, M. Foderé on | 2 | 417 |
| Carmichael, Mr. case of amputation at the hip-joint | 2 | 511 |
| Carmichael, Mr. on strangulated hernia | 4 | 340 |
| Carmichael, Mr. on phrenology | 4 | 868 |
| Carotid aneurism | 2 | 870 |
| Carotid artery, wound and ligature of | 4 | 334 |
| Carotids, effects of pressure on | 3 | 27 |
| Carson, Dr. on phthisis | 3 | 183 |
| Carter, Dr. on climate in phthisis | 1 | 380 |
| Carter, Dr. on epilepsy | 4 | 700 |
| Carter, Dr. on diabetes | 4 | 935 |
| Cartilages, ulceration of | 2 | 425 |
| Cas rares, or remarkable cases | 4 | 295 |
| Castaing, Dr. his case considered | 4 | 968 |
| Cataract, Mr. Travers on | 2 | 380 |
| Cataract, Dr. Weller on | 2 | 642 |
| Catarrhus vesicæ, observations on | 3 | 769 |
| Catarrhus vesicæ | 4 | 749 |
| Cathartics, Dr. Chapman on | 4 | 72 |
| Celsus, surgical precepts of | 3 | 68 |
| Cephalitis, Dr. Blicke on | 3 | 178 |
| Cephalonia, plague at | 2 | 580 |
| Ceylon, Mr. Marshall on | 3 | 53 |
| Chagrin, influence of, on the liver | 3 | 202 |
| Chancre, Mr. Bacot's observations on | 2 | 608 |
| Chapman, Dr. on therapeutics | 4 | 61, 551 |
| Cheltenham, mineral waters of | 1 | 600 |
| Cheltenham, Dr. M'Cabe on the waters of | 1 | 649 |
| Chemistry, Dr. Ure's dictionary of | 2 | 315 |
| Chest, wounds of, Larrey on | 1 | 446 |
| Chest, wounds of, by Baron Larrey | 2 | 257 |
| Chest, pathological examination of | 2 | 562 |
| Chest, Drs. Laennec and Forbes on | 2 | 795 |
| Chevalier, Mr. on relaxed rectum | 1 | 465 |
| Chevalier, Mr. Hunterian oration | 2 | 220 |
| Cheyne, Dr. on dysentery | 4 | 136 |
| Children, bones bent of | 2 | 441 |
| Chisholm, Dr. on tropical climates | 3 | 100, 376 |
| Chlorine, Sir H. Davy, on | 2 | 317 |
| Chlorine, Mr. Wallace on | 3 | 510 |
| Cholera, Dr. Jameson and Mr. Corbyn on the | 1 | 393 |
| Cholera of India, Mr. Boyle on | 2 | 85 |
| Cholera epidemica | 4 | 927 |
| Cholmeley, Dr. unusual case of disease | 1 | 379 |
| Chorea, remarkable case of | 3 | 659 |
| Christison, Dr. on oxalic acid | 4 | 201 |
| Chronic hepatitis, Dr. Chisholm on | 3 | 119 |
| Churchill, Mr. on acupuncture | 2 | 431 |
| Cinchonine, chemical and medical properties of | 2 | 670 |
| Circulation, physiology of | 4 | 38 |
| Circulation of the blood | 4 | 966 |
| Clark, Dr. Letter to Tommasini | 3 | 151 |
| Clarke, Dr. Mansfield, on female diseases | 2 | 757 |
| Clarke, Mr. on discharges of females, | 3 | 77 |
| Clarke, Mr. on female diseases | 4 | 635 |
| Clavicle, dislocations of | 3 | 839 |
| Climate in phthisis, Dr. Carter on | 1 | 380 |
| Climate of West Indies, Dr. Chisholm on | 3 | 100 |
| Clinical school of Edinburgh | 3 | 153 |
| Clutterbuck, Dr. case of cardiac disease | 3 | 570 |
| Clysma, article on, by M. Barbier | 2 | 348 |
| Coagulum of blood in the bladder | 4 | 316 |
| Coal tar, how far a cause of foul air, | 4 | 996 |
| Coates, Mr. case of aneurism | 2 | 870 |
| Coindet on iodine | 3 | 757 |
| Colchicum, medical properties of | 1 | 319 |
| Colchicum seeds, where to be procured | 2 | 437 |
| College of Physicians | 4 | 386 |
| College of Surgeons | 4 | 390 |
| Colleges of Physicians & Surgeons, their laws | 2 | 734 |
| Colles, Mr. on fractured cervix femoris | 1 | 482 |
| Colles, Dr. on dissection-wounds | 4 | 95 |
| Colon, disease of, simulating aneurism of the heart | 4 | 468 |

| | Vol. | Page |
|---|---|---|
| Colon, stricture of | 4 | 451 |
| Combe, Mr. George, on phrenology, | 4 | 848, 855 |
| Conception, extra-uterine | 2 | 425 |
| Conjunctiva, diseases of | 2 | 373 |
| Conquest, Dr. outlines of midwifery, | 2 | 47 |
| Constipation, Dr. Maxwell on | 4 | 937 |
| Constipation, Dr. Chisholm on | 4 | 941 |
| Constitutional symptoms of syphilis, | 2 | 611 |
| Constriction of the sphincter ani | 3 | 328 |
| Consumptive diseases, Dr. Abercrombie on | 1 | 751 |
| Contagion of plague, how long dormant | 2 | 583 |
| Contagion of plague, laws of | 2 | 584 |
| Contagion of yellow fever, Dr. Musgrave on | 4 | 979 |
| Conversion of diseases | 3 | 32 |
| Convulsions, puerperal, Dr. Dewees on | 1 | 128 |
| Conwell, Mr. on croton oil | 3 | 186 |
| Cooke, Dr. on nervous diseases | 1 | 1 |
| Cooke, Dr. on palsy | 1 | 709 |
| Cooke, Mr. his abridgment of Morgagni | 3 | 546 |
| Cooke, Dr. on epilepsy | 4 | 119 |
| Cooper, Sir A. on hernia cerebri | 2 | 251 |
| Cooper, Sir A. his lectures | 2 | 736 |
| Cooper, Sir A. on extraction of calculi | 2 | 874 |
| Cooper, Sir A. *removal of a tumor* | 2 | 880 |
| Cooper, Sir A. on dislocations, &c. | 3 | 612, 832 |
| Cooper, Sir Astley, on dilatation of the urethra | 4 | 193 |
| Cooper, Sir Astley, on dislocations | 4 | 225 |
| Cooper, Sir Astley, on fractures | 4 | 707 |
| Cooper, Sir Astley, on extraction of calculi | 4 | 803 |
| Corbyn, Mr. on the cholera | 1 | 393 |
| Corfu, plague at | 2 | 576 |
| Cornea, diseases of | 2 | 374 |
| Cornish, Mr. on dislocated femur | 3 | 180 |
| Cornwall, climate of, Dr. Forbes on the | 1 | 679 |
| Corroding ulcer of the uterus | 3 | 81 |
| Corrosive sublimate, poisoning by | 1 | 493 |
| Countenance, signs of, in disease | 4 | 813 |
| Counter-irritation | 3 | 429 |
| Coyne, Mr. on the nit. mur. acid bath | 3 | 510 |
| Crampton, Dr. on epidemic fever | 1 | 139 |
| Crampton, Dr. Philip, on leeching internal surfaces | 4 | 313 |
| Craniology, remarks on | 3 | 245 |
| Crichton, Sir Alexander, on phthisis, | 4 | 102 |
| Crichton, Dr. on yellow fever | 4 | 1002 |
| Cross, Mr. on the variolous epidemic in Norwich | 1 | 300 |
| Croton, oil of, a new old remedy | 2 | 428 |
| Cubebs, Mr. Jeffreys on, in gonorrhœa | 2 | 271 |
| Cubebs in gonorrhœa | 4 | 216 |
| Cuming, Dr. on disease of the heart, | 4 | 331 |
| Cutaneous diseases, treatment of | 1 | 490 |
| Cutaneous diseases, Mr. Wilkinson on | 4 | 132 |
| Cusack, Dr. ligature of the carotid | 4 | 334 |
| Cuvier on organization | 3 | 238 |
| Cynanche laryngea, severe cases of | 2 | 516 |
| Cynanche cellularis | 3 | 644 |
| Cystitis, Mr. Liston's case of | 3 | 681 |

D

| | Vol. | Page |
|---|---|---|
| Dasher transport, fever in | 4 | 994 |
| Davies, Dr. on the preparations of mercury | 1 | 684 |
| Davy, Dr. J. on post-mortem changes | 1 | 122 |
| Davy, Dr. on pneumo-thorax | 4 | 922 |
| Death, sudden | 2 | 389 |
| Death, sudden and mysterious | 4 | 920 |
| Delirium tremens | 4 | 918 |
| Determination of blood, what? | 3 | 18 |
| Determination defective, what? | 3 | 36 |
| Dew, Dr. Ure on | 2 | 318 |
| Dewees, Dr. on puerperal convulsions | 1 | 128 |
| Dewees, Dr. on difficult parturition | 1 | 229 |
| Dewees, Dr. on dysmenorrhœa | 1 | 479 |
| Dewees, Dr. on gastrotomy | 2 | 278 |
| Diabetes, remarks on | 3 | 265 |
| Diabetes mellitus | 4 | 200, 345 |
| Diabetes | 4 | 935 |
| Diagnosis between hydrocephalus and worm fever | 2 | 470 |
| Diagnosis, Dr. Hall on | 4 | 811 |
| Diaphoretics, observations on | 4 | 76 |
| Diaphragmatic pleuritis | 4 | 912 |
| Dictionary of chemistry | 2 | 315 |
| Diet in indigestion | 2 | 297 |
| Digestive organs, mucous membrane of | 2 | 1 |
| Digestive organs, Dr. Thomas on | 2 | 348 |
| Digitalis, on the infusion of | 1 | 494 |
| Digitalis, over-dose of | 3 | 425 |
| Dilator, Mr. Arnott's | 1 | 727 |
| Diseases, verminous, Brera on | 1 | 549 |
| Dislocations, Sir Astley Cooper on | 3 | 612, 832 |
| Dislocations, Sir Astley Cooper on | 4 | 225 |
| Dissections, wounds in | 4 | 95 |
| Distortions, spinal, Mr. Ward on | 3 | 746 |
| Distortions of the spine, Mr. Shaw on | 4 | 882 |
| Diuretics, Dr. Chapman on | 4 | 74 |
| Doctrine, new Italian, delineated | 2 | 745 |
| Douglas, Dr. on the hour-glass contraction of the uterus | 1 | 631 |
| Douglas, Dr. on puerperal fever | 4 | 83 |
| Douglas, Dr. on extraction of the placenta | 4 | 961 |
| Dracunculus, Dr. Scott | 4 | 182 |
| Drink in indigestion | 2 | 298 |
| Dropsy, final causes of | 3 | 16 |
| Dropsy, curious termination of | 3 | 53 |
| Dropsy, inflammatory, case of | 3 | 200 |
| Dropsy, renal, curious cases of | 3 | 653 |

| | Vol. | Page |
|---|---|---|
| Dublin, on the diseases of | 4 | 88 |
| Dublin Hospital Reports, Vol. III. | 4 | 313 |
| Dubois, Mr. on malignant ulcer of penis | 3 | 405 |
| Dubois, Mr. on inguinal hernia | 3 | 410 |
| Duchatelet on arachnitis | 2 | 695 |
| Dunglison on the use of the moxa | 3 | 534 |
| Dunn, Mr. on amputation of the tarsus | 2 | 873 |
| Dunn, Mr. on compound fractures | 3 | 669 |
| Duodenum, Dr. Yeats on the | 1 | 623 |
| Dupuytren, M. on fractures of the fibula | 1 | 60 |
| Dupuytren, M. on prolapsus ani | 4 | 191 |
| Dyett, Dr. on yellow fever | 4 | 1002 |
| Dysentery, treatment of | 3 | 63 |
| Dysentery, Dr. Chisholm on | 3 | 112 |
| Dysentery, Drs. Cheyne & O'Brien on | 4 | 136 |
| Dyspepsia, nature of | 3 | 21 |
| Dyspepsia, congenital | 3 | 407 |
| **E** | | |
| Ear, Mr. Swan on | 2 | 873 |
| Earle, Mr. on the meatus auditorius, | 1 | 437 |
| Earle, Mr. H. on renal calculi | 2 | 436 |
| Earle, Mr. H. on the urethra | 2 | 858 |
| Earle's, Mr. H. galvanic experience | 3 | 608 |
| Earle, Mr. on ununited fractures | 4 | 192 |
| Earle's, Mr. H. observations in surgery | 4 | 707 |
| Earle, Mr. on local irritation | 4 | 793 |
| Economy, female, Dr. Power on. | 2 | 409 |
| Ectropion, Mr. Guthrie on | 4 | 437 |
| Edinburgh medical school defended | 3 | 151 |
| Edinburgh surgical school, remarks on | 3 | 207 |
| Editor's case of hæmato-scrofulous tumor | 2 | 514 |
| Education, medical, Mr. Alcock on | 4 | 521 |
| Elaterium, Drs. Clutterbuck & Paris on | 1 | 90 |
| Elliotson, Dr. on the prussic acid | 1 | 639 |
| Ellis', Mr. biography of Dr. Gordon, | 4 | 29 |
| Elm bark, Mr. Jeffreys on | 3 | 544 |
| Emetic tartar, Mr. Jeffreys on | 3 | 539 |
| Emetics in apoplexy | 1 | 25 |
| Emetics in spasmodic diseases | 2 | 282 |
| Emetics in constipation | 3 | 410 |
| Emetics, Dr. Chapman on | 4 | 69 |
| Emphysema, remarkable case of | 2 | 238 |
| Emphysema, idiopathic, cases of | 2 | 335 |
| Empyema, Larrey on | 1 | 446 |
| Enteric mania, description of | 3 | 291 |
| Enteritis, Mr. Wade on | 4 | 193 |
| Enteritis, M. Tacheron on | 4 | 743 |
| Entropium, Mr. Guthrie on | 4 | 429 |
| Epigastrium, contusions on | 2 | 662 |
| Epilepsy, remarks on | 3 | 28 |
| Epilepsy, Dr. Prichard on | 3 | 127 |
| Epilepsy, Morgagni on | 3 | 554 |
| Epilepsy, Dr. Cooke on | 4 | 119 |
| Epilepsy, Baron Larrey on | 4 | 465 |
| Epilepsy, Dr. Carter on | 4 | 700 |
| Erethism of the brain, Dr. Nicholl on | 2 | 361, 527 |
| Eruptions, artificial, Dr. Jenner on | 3 | 89 |
| Eruptive diseases, Dr. Robinson on | 2 | 335 |
| Erysipelas treated with ung. hydrarg. | 1 | 494 |
| Erysipelas, Dr. Dickson on | 1 | 745 |
| Erysipelas, Mr. James on | 2 | 857 |
| Esophagus, stricture of | 4 | 237 |
| Esquirol on insanity | 1 | 237 |
| Esquirol on puerperal mania | 3 | 269 |
| Esquirol on suicide | 4 | 1 |
| Etiology of indigestion | 2 | 292 |
| Eudiometer, a new one | 2 | 319 |
| Evans, Mr. on the genital organs | 1 | 28 |
| Evans, Mr. on mal-practices in medicine | 4 | 768 |
| Excrescence, cauliflower | 2 | 768 |
| Experiments on the digestion of rabbits | 2 | 311 |
| Extirpation of the thyroid gland | 4 | 702 |
| Extravasation within the cranium | 4 | 952 |
| Eye, Dr. Vetch's treatise on the | 1 | 652 |
| Eye, Dr. Weller's treatise on | 2 | 626 |
| Eye, lectures on, Mr. Guthrie | 4 | 427 |
| Eyes, diseases of, Mr. Travers on | 2 | 105, 369 |
| **F** | | |
| Falret, Dr. on suicide | 4 | 1 |
| Farr, Mr. on scrofula | 1 | 119 |
| Farr, Mr. on cancer | 2 | 732 |
| Faulkner, Sir A. B. on the plague | 1 | 570 |
| Females, on the diseases of | 4 | 635 |
| Femoral aneurism, spontaneous cure of | 4 | 955 |
| Femoro-coxalgia, moxa in | 3 | 530 |
| Femur, fracture, with separation of condyles | 2 | 242 |
| Femur, on dislocation of | 3 | 180 |
| Ferguson, Dr. on marsh poison | 2 | 585 |
| Fever, epidemic, Drs. Grattan and Crampton on | 1 | 139 |
| Fever, Mr. H. Sandwith on | 2 | 203 |
| Fever, Dr. Reid on | 2 | 327 |
| Fever, congestive, Prosper Alpinus on | 2 | 685 |
| Fever, yellow, Dr. Chisholm on | 3 | 109 |
| Fever, Dr. Armstrong on | 3 | 393 |
| Fever, the various theories of | 3 | 818 |
| Fever, bilious remittent | 4 | 211 |
| Fever, yellow, Dr. O'Halloran | 4 | 282 |
| Fever, yellow, in the Bann and at Ascension | 4 | 916 |
| Fevers of Spain, Dr. Jackson on | 2 | 485 |
| Fielding, Mr. on fractured patella | 3 | 179 |
| Fits, Dr. Latham's treatment of | 1 | 613 |
| Fleurens, M. on the nervous system, | 4 | 705 |
| Fœtus, spontaneous evolution, Dr. Gooch on | 1 | 611 |
| Fœtus, crying of, in utero | 3 | 221 |
| Fœtus, extracted post mortem | 3 | 677 |
| Fœtus, destruction of brain in | 4 | 800 |
| Fœtus, venereal disease in | 4 | 839 |

| | Vol. | Page |
|---|---|---|
| Folloon, Dr. on diseases of the heart, | 2 | 532 |
| Food, adulteration of .......... | 4 | 599 |
| Forbes, Dr. on the climate of Cornwall .................... | 1 | 679 |
| Forbes, Dr. translation of Laennec.. | 2 | 795 |
| Forbes, Dr. on variolous epidemic .. | 3 | 646 |
| Forbes, Dr. James, on tar-vapour .. | 3 | 651 |
| Forensic medicine, Dr. Smith on.... | 2 | 387 |
| Forensic case, Mr. Charles Bell's... | 2 | 561 |
| Fosbroke, Mr. case of scrofula ..... | 3 | 671 |
| Fractured cervix femoris, Mr. Colles on ................ | 1 | 482 |
| Fractures of the fibula, M. Dupuytren on .................... | 1 | 60 |
| Fractures of the cervix femoris .... | 3 | 628 |
| Fractures, compound, Mr. Dunn on, | 3 | 669 |
| Fractures of the thigh, Mr. Amesbury on.................. | 3 | 915 |
| Fractures, ununited, Mr. Earle on .. | 4 | 192 |
| Fractures of the cranium (Mr. Baker) | 4 | 953 |
| Fractures, diagnosis of........... | 4 | 954 |
| Frost-bites, treatment of......... | 2 | 246 |
| Fucus helminthocorton, Mr. Farr on, | 2 | 732 |
| Fumigation, sulphureous, Mr. Wallace on .................... | 2 | 570 |
| Fungus hæmatodes retinæ ........ | 2 | 653 |
| **G** | | |
| Galvanic experiments on digestion .. | 2 | 311 |
| Gangrene, traumatic, amputation during ...................... | 2 | 187 |
| Gangrene, Dr. Grattan's case of.... | 2 | 521 |
| Gasc, M. on peritonitis .......... | 1 | 161 |
| Gastritis, acute and chronic ....... | 2 | 6 |
| Gastrotomy, cases of ............ | 1 | 101, 103 |
| Gastrotomy, Dr. Dewees on ...... | 2 | 278 |
| Geneva, salubrity of............... | 3 | 383 |
| Genital organs, Mr. Evans on the .. | 1 | 28 |
| Georget, Dr. on mania............ | 3 | 701 |
| Gibney, Dr. on oil of turpentine.... | 3 | 406 |
| Glasgow Lunatic Asylum... ...... | 4 | 775 |
| Glaucoma, Dr. Weller on ........ | 2 | 646 |
| Glottis, on œdema of the.......... | 4 | 452 |
| Glysters, observations on ......... | 2 | 353 |
| Goitre, connected with cardiac disease ........................ | 3 | 19 |
| Golding, Dr. case of arteritis ...... | 2 | 439 |
| Golis, Dr. on hydrocephalus acutus, | 2 | 465 |
| Gonorrhœa, Mr. Jeffrey's on ...... | 2 | 271 |
| Gooch, Dr. on the spontaneous evolution of the fœtus .......... | 1 | 611 |
| Gooch, Dr on puerperal insanity .. | 1 | 615 |
| Gooch, Dr. on uterine hæmorrhage, | 3 | 662 |
| Good, Dr. J. M. study of medicine.. | 3 | 574, 806 |
| Good, Dr. study of medicine ....... | 4 | 155 |
| Goodisson, Mr. on the Ionian Islands, | 4 | 211 |
| Gordon, Dr. biography of ......... | 4 | 29 |
| Gout, a salutary process .......... | 3 | 38 |
| Gozo, plague at ................. | 2 | 575 |
| Graham, Dr. on dropsy ........... | 3 | 200 |

| | Vol. | Page |
|---|---|---|
| Granville, Dr. on ovario-gestation.. | 1 | 469 |
| Granville, Dr. on the prussic acid .. | 1 | 686 |
| Grattan, Dr. on epidemic fever .... | 1 | 139 |
| Grattan, Dr. case of gangrene...... | 2 | 521 |
| Gravel, Dr. Prout on ............ | 2 | 89 |
| Gregory, Dr. on apoplexy ........ | 1 | 25 |
| Gregory, Dr. on enteritis.......... | 1 | 167 |
| Gregory's, Dr. George, practice of physic .................... | 1 | 419 |
| Gregory, Professor, his death ...... | 2 | 216 |
| Gregory, Dr. on marasmus ........ | 2 | 868 |
| Gregory, Dr. on malformation of the heart .................. | 2 | 871 |
| Gregory, Dr. on cynanche cellularis, | 3 | 644 |
| Gregory, Dr. George, on post-vaccine disease ................ | 4 | 806 |
| Gregory's, Dr. George, case of encysted tumor................ | 4 | 915 |
| Guadiana, medical topography of .. | 2 | 588 |
| Gun-shot wounds, Messrs. Guthrie, Hennen, Laurent, and Percy on | 2 | 165 |
| Guthrie, Mr. on artificial pupil .... | 1 | 71 |
| Guthrie, Mr. operative surgery of the eye .................... | 4 | 427, 658 |
| **H** | | |
| Haden, Mr. on the poison of food.... | 4 | 765 |
| Hæmatemesis .................. | 4 | 928 |
| Hæmoptysis .................... | 4 | 157 |
| Hæmorrhage, internal, case of .. .. | 2 | 668 |
| Hæmorrhage, final causes of ....... | 3 | 16 |
| Hæmorrhage, internal .......... . | 4 | 930 |
| Hæmorrhoids, Dr. Good on........ | 3 | 588 |
| Halford, Sir H. on symptomatology, | 1 | 634 |
| Hall, Dr. M. on puerperal disease .. | 1 | 195 |
| Hall, Dr. Marshall, cases by ....... | 3 | 387 |
| Hall, Dr. on semeiology .......... | 4 | 811 |
| Hamilton, Dr. on mercury ........ | 1 | 41 |
| Hammond, Mr. on destroyed brain in a fœtus .................. | 4 | 800 |
| Hancock, Dr. on pestilence ........ | 2 | 569 |
| Hare-lip, double, curious case of.... | 3 | 433 |
| Harrison, Dr. on the pathology of eruptive fevers .............. | 1 | 325 |
| Harrison, Dr. his case of hæmorrhage | 2 | 334 |
| Harrison, Mr. on syphilitic eruptions | 2 | 598 |
| Harrowgate, mineral waters of ..... | 1 | 595 |
| Hartle, Dr. inspector, his reports on yellow fever.............. | 4 | 993 |
| Harvey on life .................. | 3 | 251 |
| Harvey, curious preparations of.... | 4 | 234 |
| Hawkins, Mr. on ulcerations of the larynx .................. | 4 | 471 |
| Haslam, Dr. on sound mind ...... | 1 | 237 |
| Hastings, Dr. on the mucous membrane of the lungs.......... .. | 1 | 325 |
| Head, on wounds of, by Baron Larrey | 2 | 249 |
| Healey, Mr. on a paralytic affection, | 4 | 318 |
| Heart, curious case of diseased .... | 1 | 442 |
| Heart, diseased, case of .......... | 1 | 746 |
| Heart, diseases of, Dr. Reeder...... | 2 | 401 |
| Heart, diseases of, Dr. Fodéré...... | 2 | 417 |

| Entry | Vol. | Page |
|---|---|---|
| Heart, diseases of, Dr. Folloon | 2 | 532 |
| Heart, venereal affection of | 3 | 413 |
| Heart, remarkable disease of | 3 | 563 |
| Heart, disease of, Dr. Cuming | 4 | 331 |
| Heart, chronic inflammation of | 4 | 548 |
| Heart, enlarged | 4 | 706 |
| Helminthia, Dr. Good on | 3 | 585 |
| Helminthocorton, or Corsican moss, | 2 | 732 |
| Hennen, Dr. on military surgery | 1 | 283 |
| Hepatic abscess from wounds of head | 2 | 253 |
| Hepatic functions, Drs. Pearson and M'Donnel on | 2 | 281 |
| Hepatic dysentery | 3 | 113 |
| Hepatic epilepsy | 3 | 146 |
| Hepatitis, Dr. Chisholm on | 3 | 115 |
| Hepato-pulmonic disease | 3 | 675 |
| Hernia, Dr. Yeats on | 1 | 373 |
| Hernia cerebri, treatment of | 2 | 251 |
| Hernia, Mr. Shaw on | 2 | 553 |
| Hernia, Mr. Lizars on | 3 | 408 |
| Hernia, Mr. Lizars on the treatment of | 4 | 232 |
| Herpes preputialis, Mr. Evans on | 1 | 31 |
| Hey, Mr. his life, by Mr. Pearson | 4 | 827 |
| Hill, Mr. on tic douloureux | 3 | 176 |
| Hip-joint, amputation at | 2 | 194 |
| Hip-joint, operation, Mr. Carmichael, | 2 | 511 |
| Hip-joint, luxation of | 3 | 180, 415 |
| Hip-joint, scrofula of | 3 | 340 |
| Hip-joint dislocation, Sir A. Cooper, | 3 | 620 |
| Hip-joint, dislocations of | 4 | 342 |
| Hippocrates, critique on | 3 | 270 |
| Homicide | 4 | 607 |
| Homicide, trial for | 4 | 969 |
| Hooping-cough, Mr. Robertson on | 1 | 754 |
| Hooping-cough, new theory of | 4 | 449 |
| Hordeolum, treatment of | 2 | 383 |
| Hordeolum | 2 | 632 |
| Hospital reports, (Dublin), Vol. III. | 4 | 77 |
| Howship, Mr. on the diseases of the rectum | 1 | 222 |
| Howship, Mr. obstructed œsophagus, | 4 | 317 |
| Howship, Mr. on the urinary organs, | 4 | 369 |
| Human combustion | 4 | 604 |
| Hume, Dr. on tonic treatment of phthisis | 4 | 189 |
| Hunter on life | 3 | 252 |
| Hunter, John, remarks on his death, | 3 | 568 |
| Hunterian oration, Mr. Chevalier's | 2 | 220 |
| Hurricane, Sir Gilbert Blane on | 3 | 743 |
| Hutchinson on tic douloureux | 1 | 111 |
| Hutchinson, Dr. on infanticide | 1 | 428 |
| Hutchinson, Mr. on neuralgia | 3 | 495 |
| Hutchison, Mr. on bronchocele | 2 | 866 |
| Hydatid, Mr. Pretty's case | 4 | 930 |
| Hydatids of the uterus | 2 | 774 |
| Hydrocephalus acutus, Dr. Golis on, | 2 | 465 |
| Hydrocephalus, Sir G. Blane on | 2 | 669 |
| Hydrocyanic acid, Dr. Elliotson on, | 1 | 639 |
| Hydrocyanic acid, Dr. Granville on, | 1 | 686, 787 |
| Hydrophobia, M. Majendie on | 1 | 763 |
| Hydrophobia | 3 | 429 |
| Hydrophobia, Morgagni on | 3 | 559 |
| Hydrophobia, Marochetti on | 4 | 917 |
| Hydrophobia, supposed case of | 4 | 942 |
| Hydrophthalmia, Dr. Weller on | 2 | 647 |
| Hydrothorax, case of | 1 | 458 |
| Hydrothorax, pathology of | 2 | 803 |
| Hydrothorax, M. Tacheron on | 4 | 757 |
| Hygiene, military | 1 | 125 |
| Hygiene, tropical | 3 | 105 |
| Hypochondriasis, curious case of | 3 | 667 |
| Hypochondriasis | 4 | 162 |
| Hypopion | 2 | 375 |
| Hysteritis, Mr. Clarke on | 2 | 763 |
| **I** | | |
| Icterus, pathology of | 3 | 589 |
| Iliac artery, ligature of | 1 | 758 |
| Iliac, external, propriety of tying | 2 | 270 |
| Iliac artery, on the ligature of | 2 | 556 |
| Iliac artery, obliteration of | 3 | 431 |
| Indigestion, Dr. Philip on | 2 | 284, 862 |
| Infanticide, Dr. Hutchinson on | 1 | 428 |
| Infanticide, trial for | 4 | 972 |
| Infants, syphilis in | 1 | 47 |
| Infants, on the vitality of | 4 | 738 |
| Inflammation of arteries | 2 | 50 |
| Inflammation of the spinal arachnoid, | 2 | 724 |
| Inflammation, Mr. James on | 2 | 834 |
| Inflammation, final causes of | 3 | 16 |
| Inflammation, remarkable case of | 3 | 197 |
| Inflammation, Dr. Lucas on | 4 | 38 |
| Inflammation, products of | 4 | 223 |
| Inflammation of the diaphragmatic pleura | 4 | 912 |
| Inflammations, cutaneous | 4 | 489 |
| Injecting machine for the stomach | 4 | 742 |
| Insanity, Drs. Haslam, Burrows, and Esquirol on | 1 | 237 |
| Insanity, puerperal, Dr. Gooch on | 1 | 615 |
| Insanity, traits of, in Bedlam | 4 | 511 |
| Intermittents, masked | 2 | 450 |
| Internal surfaces, leeches to | 4 | 313 |
| Intestine, portion of, expelled | 4 | 932 |
| Intestines, painful affections of the | 1 | 386 |
| Iodine, Dr. Coindet on, in bronchocele | 2 | 322 |
| Iodine, Dr. Brera, &c. on | 3 | 757 |
| Ionian islands, Mr. Goodisson on | 4 | 211 |
| Ireland, Dr. case of emphysema | 2 | 335 |
| Iris, on the powers of the | 3 | 533 |
| Iritis, Dr. Weller on | 2 | 648 |
| Iritis, Dr. Smith on | 3 | 666 |
| Irritability, inordinate, Mr. Wardrop on | 1 | 485 |
| Irritation, Sir Astley Cooper on | 2 | 737 |
| Irritation, Mr. Earle on | 4 | 793 |
| Ischuria, Sir Astley Cooper on | 2 | 744 |
| Italy, present state of medicine in | 2 | 745 |

| | Vol. | Page |
|---|---|---|
| **J.** | | |
| Jackson, Dr. on the Andalusian fevers | 2 | 485 |
| James, Mr. on inflammation ....... | 2 | 834 |
| Jameson, Dr. on the cholera morbus, | 1 | 393 |
| Jaundice, remarkable case of ...... | 3 | 94 |
| Jaundice, Dr. Henry Marsh on.. .. | 4 | 319 |
| Jaw, on injury of, Mr. Powell...... | 4 | 766 |
| Jeffreys, Mr. Henry, on cubebs .... | 2 | 271 |
| Jeffreys', Mr. cases in surgery...... | 3 | 535 |
| Jenner, Dr. on vaccination ........ | 1 | 702 |
| Jenner, Dr. his circular to the profession .................... | 1 | 764 |
| Jenner, Dr. on artificial eruptions .. | 3 | 89 |
| Johnson, Dr. his reply to certain reviewers .................. | 1 | 535 |
| Johnson, Dr. James, on tropical hygiene ..... ............... | 3 | 105 |
| Johnson, Dr. James, case of visceral disease .................. | 3 | 456 |
| Johnson, Dr. James, on renal dropsy, | 3 | 654 |
| Johnson, Dr. James, case of puerperal fever............ ...... | 3 | 650 |
| Johnson, Dr. James, case of Mr. Knox | 3 | 694 |
| Johnson, Dr. on disease of the heart, | 4 | 548 |
| Joints, hip, dislocations of ........ | 4 | 707 |
| Journal of phrenology ............. | 4 | 847 |
| Journals, medical, increase of...... | 4 | 911 |
| Jurisprudence, medical............ | 4 | 383, 596 |
| **K.** | | |
| Kandyan country, topography of .. | 3 | 55 |
| Keate, Mr. case of bony tumor .... | 1 | 98 |
| Kennedy, Dr. J. case of arteritis.... | 2 | 60 |
| Kennedy, Dr. on tetanus.......... | 3 | 423 |
| Kentucky, university of .......... | 2 | 219 |
| Kergaradec on the stethoscope .... | 3 | 661 |
| Kerrison, Dr. on tic douloureux.. .. | 2 | 63 |
| Kerrison, Dr. on dilatation of the urethra .................... | 4 | 802 |
| Kidney, dropsy of.............. .. | 3 | 653 |
| Kinine, the medical properties of ... | 2 | 670 |
| Knee, dislocations of.............. | 3 | 832 |
| Knife-eater, account of .......... | 4 | 218 |
| **L.** | | |
| Labour, difficult case of .......... | 2 | 438 |
| Labour, premature, Mr. Barlow on.. | 3 | 319 |
| Lacon, author of, remonstrated with, | 2 | 522 |
| Lachrymal passage, obstruction of... | 2 | 385 |
| Lachrymal gland, diseases of ...... | 4 | 344 |
| Laennec, Dr. on thoracic diseases .. | 2 | 795 |
| Laird's, Dr. case of hooping-cough.. | 3 | 131 |
| Lallemand, M. on the pathology of the brain ............ ..... | 3 | 465 |
| Lancet, (Medical Journal,) examined, | 4 | 973 |
| Larrey, Baron, on wounds of the chest ...................... | 1 | 446 |
| Larrey, Baron, on wounds of the intestines .................. | 1 | 473 |
| Larrey, Baron, his Russian campaign, | 2 | 237 |
| Larrey, Baron, surgical memoirs.... | 3 | 523 |

| | Vol. | Page |
|---|---|---|
| Larrey, Baron, on epilepsy ........ | 4 | 465 |
| Laryngeal disease, Mr. Shaw on.... | 4 | 188 |
| Larynx, œdema of................ | 4 | 452 |
| Larynx, syphilitic ulcers of........ | 4 | 471 |
| Latham, Dr. on the solanum tuberosum.................... | 1 | 384 |
| Latham, Dr. on bleeding in fits .... | 1 | 613 |
| Lavemens, on the use of ..... .... | 2 | 353 |
| Lawrence, Mr. remarks on ........ | 3 | 239 |
| Leamington, mineral waters of .... | 1 | 603 |
| Lecturers, their privileges discussed, | 4 | 973 |
| Lectures, Sir Astley Cooper's...... | 2 | 736 |
| Leeches to internal surfaces........ | 4 | 313, 940 |
| Leeches, Dr. Price on ........... | 4 | 697 |
| Leeching, observations on ........ | 2 | 357 |
| Legal medicine .................. | 4 | 968 |
| Lendrick, Dr. case of poisoning .... | 2 | 534 |
| Life, Dr. Barclay on............... | 3 | 225 |
| Ligature of arteries, described by Celsus ..................... | 3 | 68 |
| Lime, muriate of, Dr. Hamilton on.. | 1 | 53 |
| Lip, cancer of, Dr. Bull on........ | 4 | 464 |
| Lippitudo ...................... | 2 | 384 |
| Lips, on diseases of .............. | 4 | 794 |
| Liquor morphii citratis, preparation of | 1 | 127 |
| Lisbon, medical topography of...... | 2 | 589 |
| Liston, Mr. his letters respecting the Infirmary of Edinburgh.... | 3 | 207 |
| Liston, Mr. case of cystitis ........ | 3 | 681 |
| Lithontriptics, Dr. Chapman on .... | 4 | 75 |
| Lithotomy, new method of operating, | 2 | 337 |
| Lithotomy, Mr. Martineau on...... | 2 | 877 |
| Lithotomy, Mr. Barlow on ........ | 3 | 65 |
| Lithotomy remarkable case of...... | 3 | 73 |
| Liver, remarkable disease of ...... | 3 | 694 |
| Liver-cough, Dr. Brooke's case of .. | 2 | 524 |
| Lizars, Mr. on hernia ............ | 3 | 408 |
| Lizars, Mr. on the treatment of hernia | 4 | 232 |
| Lloyd, Mr. on scrofula............ | 3 | 156, 333 |
| Local irritation, Mr. Earle on ...... | 4 | 793 |
| Lucas, Dr. on inflammation and fever | 4 | 38 |
| Lucretius, remarks on............ | 3 | 232 |
| Lunacy, destructive propensities in.. | 4 | 781 |
| Lunatic Asylum of Glasgow ...... | 4 | 775 |
| Lungs, inflammation of, from wounds of other parts ........ | 2 | 192 |
| Luscombe, Dr. on the health of soldiers ...................... | 1 | 125 |
| **M** | | |
| M'Cabe, Dr. on the waters of Cheltenham .................... | 1 | 649 |
| M'Donnel, Dr. on the hepatic functions ........... .......... | 2 | 281 |
| M'Keever, Dr. case of ruptured vagina | 2 | 530 |
| Mackenzie, Dr. on mineral waters .. | 1 | 589 |
| Macmichael, Dr. on scarlet fever.... | 3 | 724 |
| Magnetism, animal .............. | 3 | 255 |
| Majendie, M. on hydrophobia .... | 1 | 763 |
| Majendie, Dr. on chorea .......... | 3 | 659 |

| | Vol. | Page |
|---|---|---|
| Majendie, Dr. on the spinal marrow, | 4 | 702 |
| Malaria in St. James's Park.......... | 3 | 422 |
| Malta, plague at ................ | 2 | 574 |
| Malvern, mineral waters of........ | 1 | 604 |
| Mammæ, on enlargement of ...... | 4 | 838 |
| Mammary scrofula .............. | 3 | 169 |
| Mammary abscess, treatment of.... | 3 | 541 |
| Mania, puerperal, M. Esquirol on .. | 3 | 269 |
| Mania, Dr. Prichard on ........... | 3 | 277 |
| Mania, M. Georget on ............ | 3 | 701 |
| Manual of anatomy, Mr. Shaw's .... | 2 | 551 |
| Marasmus, Dr. Gregory on........ | 2 | 868 |
| Marathia, plague at ............ | 2 | 577 |
| Marcet, Dr. on nephritis calculosa .. | 1 | 109 |
| Marsh poison, Dr. Ferguson on .... | 2 | 585 |
| Marsh, Dr. on jaundice ........... | 4 | 319 |
| Marsh, Dr. on diabetes............ | 4 | 345 |
| Marshall, Mr. on Ceylon .......... | 3 | 53 |
| Martineau, Mr. on lithotomy ...... | 2 | 877 |
| Materia medica, Dr. Chapman on .. | 4 | 61 |
| Materialism, Dr. Barclay on ...... | 3 | 226 |
| Materialism and immaterialism .... | 4 | 160 |
| Matlock, mineral waters of........ | 1 | 593 |
| Mayo, Mr. case of axillary aneurism, | 3 | 390 |
| Medical transactions.............. | 1 | 371 |
| Medical science in the United States | 1 | 496 |
| Medical practitioners, actions against | 4 | 391 |
| Medical jurisprudence ......... .. | 4 | 383, 596 |
| Medicine, forensic, Dr. Smith on .. | 2 | 387 |
| Medicine, the study of, Dr. Good's.. | 3 | 574 |
| Medicines, large doses of .......... | 2 | 752 |
| Medico-chirurgical transactions .... | 2 | 865 |
| Medico-chirurgical transactions, vol. xii.................... | 3 | 386 |
| Medico-chirurgical transactions .... | 4 | 790 |
| Medulla spinalis, inflammation of .. | 2 | 429 |
| Melanose, M. Breschet on ......... | 3 | 208 |
| Melena, Dr. Nicholl on............ | 2 | 529 |
| Melena, Dr. Good on ............ | 3 | 592 |
| Membranes, false, in pleurisy ...... | 2 | 798 |
| Meningine, Dr. Scoulton on ....... | 4 | 698 |
| Menstruation, suppressed, effects of | 1 | 107 |
| Menstruation, Dr. Power on ...... | 2 | 409 |
| Menstruation, deficient ........... | 2 | 759 |
| Mercurial vapour, Dr. Burnett on .. | 4 | 1010 |
| Mercury, Dr. Hamilton on ........ | 1 | 41 |
| Mercury, Dr. Davies on the preparations of .................... | 1 | 684 |
| Mercury, case of gangrene from.... | 2 | 522 |
| Mercury, various authors on ...... | 2 | 598 |
| Mercury in acute diseases ........ | 4 | 222 |
| Mercury, Mr. Swan on the action of, | 4 | 277 |
| Merriman, Dr. on difficult parturition | 1 | 510 |
| Merriman, Dr. on the vitality of infants.. .................. | 4 | 738 |
| Metastasis, curious cases of........ | 3 | 401 |
| Metastatic inflammations.......... | 3 | 641 |
| Midwifery, Burns' principles of .... | 1 | 579 |
| Midwifery, Dr. Conquest's outlines of | 2 | 47 |
| Midwifery, Dr. Ramsbotham on.... | 2 | 141 |
| Military surgery, Dr. Hennen on.... | 1 | 283 |
| Military surgery, Messrs. Hennen and Guthrie on.............. | 2 | 165 |
| Military surgery, Baron Larrey on.. | 2 | 240 |
| Millbank Penitentiary ............ | 4 | 235 |
| Millingen, Dr. his manual ....... | 1 | 125 |
| Monteath, Dr. translation of Weller on the eye................. | 2 | 626 |
| Morgagni, abridged by Cooke ...... | 3 | 546 |
| Mortality, comparative, in London.. | 3 | 737 |
| Moulson, Dr. on nervous diseases .. | 3 | 556 |
| Moxa, Baron Larrey on .......... | 3 | 523 |
| Moxa, Mr. Dunglison on .......... | 3 | 534 |
| Mucous membrane of the digestive organs ...................... | 2 | 1 |
| Mucous discharges (white) of females | 2 | 758 |
| Muscle, degeneration of .......... | 4 | 945 |
| Musgrave, Dr. on yellow fever...... | 4 | 979 |

**N**

| | Vol. | Page |
|---|---|---|
| Nephritis calculosa, Dr. Marcet on.. | 1 | 109 |
| Nerve, wounds of................ | 4 | 450 |
| Nerves, on affections of .......... | 2 | 63 |
| Nerves, Mr. Charles Bell on their functions ................... | 2 | 809 |
| Nerves, spinal, experiments on .... | 3 | 682 |
| Nerves, on painful affections of .... | 4 | 766 |
| Nervous diseases, Dr. Cooke on .... | 1 | 1 |
| Nervous sympathy, how produced .. | 2 | 295 |
| Nervous system, Mr. Bell's remarks on | 2 | 566 |
| Nervous constitution, Dr. Parry on.. | 3 | 25 |
| Nervous system, Dr. Prichard on .. | 3 | 122 |
| Nervous system, M. Fleurens on.... | 4 | 705 |
| Nervous system, physiology of ..... | 4 | 966 |
| Neuralgia, cases of .............. | 1 | 748 |
| Neuralgia, singular case of ......... | 2 | 426 |
| Neuralgia, various authors on...... | 3 | 495 |
| Neuralgia, Dr. Good on ........... | 4 | 167 |
| Nicholl, Dr. elements of pathology.. | 2 | 133 |
| Nicholl, Dr. on erethism of the brain, | 2 | 361, 527 |
| Nicholl, Dr. on melena............ | 2 | 529 |
| Nimmo, Dr. on oil of croton ...... | 3 | 186 |
| Noia, plague at, in 1815 .......... | 2 | 582 |
| Non-pulsation of arteries.......... | 3 | 566 |
| Nose, remarkable tumor on........ | 3 | 76 |
| Nostalgia, Baron Larrey on...... . | 3 | 532 |
| Nostrum medicines, Dr. Paris on.... | 1 | 89 |
| Nuisances ...................... | 4 | 419 |

**O**

| | Vol. | Page |
|---|---|---|
| O'Beirne, Dr. on tetanus.......... | 4 | 335 |
| O'Brien, Dr. on dysentery ........ | 4 | 136 |
| Observations, practical, in midwifery | 2 | 141 |
| Observations in medicine, &c. by Mr. T. Sandwith ............ | 2 | 395 |
| Obstetric delinquency ............ | 4 | 961 |
| Obstruction of the bowels, cases of.. | 3 | 51 |
| Œdema of the glottis ............ | 4 | 452 |
| Œsophagotomy................... | 4 | 947 |
| Œsophagus, stricture of .......... | 3 | 540, 677 |
| Œsophagus, case of obstructed .... | 4 | 317 |

| | Vol. | Page |
|---|---|---|
| O'Halloran on the Spanish fever.... | 2 | 817 |
| O'Halloran, Dr. on yellow fever .... | 4 | 282 |
| Oil of croton-tiglium .............. | 3 | 186 |
| Oil of turpentine, Dr. Gibney on.... | 3 | 406 |
| Omentum, protrusion of .......... | 2 | 261 |
| Ophthalmia, Prussian treatment of.. | 1 | 100 |
| Ophthalmia, scrofulous, Mr. Jeffreys | 3 | 536 |
| Ophthalmic inflammation, cure of .. | 2 | 370 |
| Ophthalmic inflammation, suppurative.................... | 2 | 372 |
| Ophthalmic hospital extinct ....... | 2 | 448 |
| Opiologia; or confessions of opium eaters .................... | 2 | 881 |
| Opium, particular preparation of .. | 2 | 219 |
| Opium, its effects on the human system ...................... | 2 | 883 |
| Opium, on poisoning by .......... | 3 | 433 |
| Opium injected into the veins...... | 4 | 935 |
| Opium in acute mania ............ | 4 | 939 |
| Organic life, Mr. Pring on ........ | 1 | 400 |
| Organization and life ............ | 3 | 225 |
| Os uteri, case of separation, by Mr. Scott ...................... | 2 | 876 |
| Osborn, Dr. on the urine.......... | 2 | 41 |
| Osteo-sarcomatous tumor ........ | 2 | 511 |
| Osteology, lectures on, by Mr. Wilson | 1 | 411 |
| Ovarian dropsy cured by operation.. | 3 | 190 |
| Ovario-gestation, Dr. Granville on.. | 1 | 469 |
| Ovum, Mr. Stanley on the ........ | 1 | 638 |
| Oxalic acid, experiments on........ | 4 | 201 |
| Oxymuriate of mercury, swallowed.. | 2 | 534 |

P

| | Vol. | Page |
|---|---|---|
| Palate, the congenital division of.... | 1 | 440 |
| Palmer, Dr. S. on neuralgia........ | 3 | 495 |
| Palpitations of the heart, Dr. Foderé on | 2 | 417 |
| Palsy, Dr. Cooke on............. | 1 | 709 |
| Panckouckerie ................... | 2 | 447 |
| Par vagum, remarks on .......... | 2 | 566 |
| Paralysis, tourniquets in .......... | 2 | 279 |
| Paralysis, (nervous,) case of ...... | 3 | 181 |
| Paralysis, Dr. Prichard on ........ | 4 | 926 |
| Paralytic affection, curious case of .. | 4 | 318 |
| Paraplegia, Dr. Baillie on.......... | 1 | 375 |
| Paris, Dr. his pharmacologia ...... | 1 | 81 |
| Paris, Dr. on medical jurisprudence.. | 4 | 383, 596 |
| Parry, Dr. elements of pathology ... | 3 | 1 |
| Parry, Dr. on the pulse............ | 4 | 38 |
| Parturition, difficult, Dr. Dewees on, | 1 | 229 |
| Parturition, Dr. Merriman on ...... | 1 | 510 |
| Patella, dislocation of ............ | 2 | 682 |
| Patella, on fracture of............. | 3 | 179 |
| Patella, fractures of .............. | 3 | 834 |
| Pathology of the urine ............ | 2 | 41 |
| Pathology, Dr. Nicholl's elements of, | 2 | 133 |
| Pathology of indigestion .......... | 2 | 296 |
| Pathology of epilepsy ............ | 3 | 129 |
| Pathology of the brain in mania .... | 3 | 282 |
| Pathology of the brain, M. Lallemand | 3 | 465 |
| Pathology, Dr. Pring on .......... | 4 | 241 |
| Pathology of the intestinal canal.... | 4 | 673 |
| Pattison, Dr. G. on aneurism ...... | 1 | 741 |
| Pearson, Dr. on apoplexy.......... | 1 | 320 |
| Pearson, Mr. his life of Mr. Hey.... | 4 | 827 |
| Pelvis, distortions of, Mr. Barlow on, | 3 | 318 |
| Pemberton on the abdominal viscera, | 1 | 161 |
| Penis, cancer of .................. | 4 | 837 |
| Penitentiary at Millbank .......... | 4 | 235 |
| Penzance, climate of, Dr. Forbes on the ........................ | 1 | 679 |
| Percival, Mr. on veterinary medicine, | 4 | 583 |
| Percussion in chronic pleurisy...... | 2 | 800 |
| Percussion, as a means of diagnosis.. | 4 | 569 |
| Pericardiac inflammation.......... | 2 | 395 |
| Perineum, ruptured .............. | 2 | 684 |
| Perineum, perforation of.......... | 4 | 318 |
| Periscope, on the importance of.... | 4 | 911 |
| Peritoneal inflammation, Mr. Guthrie on.......................... | 2 | 171 |
| Peritonitis, Drs. Broussais, Gasc, Abercrombie, Pemberton, and Montfalcon on .............. | 1 | 161 |
| Peritonitis, supposed from poison .. | 4 | 970 |
| Perivoli, plague at .............. | 2 | 578 |
| Pestilence, Dr. Hancock on........ | 2 | 665 |
| Petechiæ hæmorrhagicæ .......... | 4 | 206 |
| Pett, Dr. his death .............. | 4 | 98 |
| Phagedena, Mr. Welbank on ...... | 2 | 875 |
| Pharmaceutical formulæ .......... | 4 | 938 |
| Pharmacologia, Dr. Paris' ........ | 1 | 80, 534 |
| Philadelphia Journal of Medical and Physical Sciences .............. | 2 | 275 |
| Philip, Dr. W. on the calculous diathesis ......................... | 1 | 607 |
| Philip, Dr. on indigestion ........ | 2 | 284, 862 |
| Philip, Dr. Wilson, his physiology .. | 3 | 596 |
| Phlebitis, curious case of.......... | 2 | 453 |
| Phlegmasies chroniques, M. Broussais on ...................... | 2 | 1 |
| Phlegmatia dolens, Dr. Hosack on .. | 3 | 432 |
| Phrenological Society's Transactions, | 4 | 847 |
| Phrenological Journal ............ | 4 | 847 |
| Phrenology, Dr. Barclay on........ | 3 | 245 |
| Phrenology, observations on ...... | 3 | 896 |
| Phrenology, forensic.............. | 4 | 969 |
| Phthisis, Dr. Carson's proposal in .. | 3 | 183 |
| Phthisis, Sir A. Crichton on ...... | 4 | 102 |
| Phthisis, tonic treatment of........ | 4 | 189 |
| Phthisis pulmonalis, Mr. Gaitskell on | 4 | 929 |
| Physic, theory and practice of, by Dr. Gregory .................. | 1 | 419 |
| Physicians, Royal College of ...... | 3 | 219 |
| Physiological experiments on digestion ...................... | 2 | 311 |
| Physiological pathology .......... | 4 | 934 |
| Physiology, general principles of.... | 3 | 596 |
| Physiology of the circulation ...... | 4 | 38 |
| Pierson, Dr. on the hepatic functions | 2 | 281 |
| Pinel, M. on the medulla spinalis ... | 2 | 429 |
| Placenta, Mr. Barlow on .......... | 3 | 306 |
| Placenta, extraction of............ | 4 | 961 |

| | Vol. | Page |
|---|---|---|
| Plague, Egyptian, Dr. Webb on .... | 1 | 391 |
| Plague, Sir A. B. Faulkner on the .. | 1 | 570 |
| Plague, Mr. Tully and Dr. Hancock on | 2 | 569 |
| Plethora, Dr. Good on ............ | 4 | 156 |
| Pleurisy, pathology of ............ | 2 | 796 |
| Pleurisy, chronic ............... | 2 | 800 |
| Pleuritis, M. Tacheron on ........ | 4 | 754 |
| Plumbe, Mr. on porrigo .......... | 2 | 534 |
| Pneumo-thorax.................. | 2 | 804 |
| Pneumonia, Dr. Forbes on ........ | 4 | 928 |
| Poison, apparatus for removing .... | 3 | 683 |
| Poison of food, Mr. Haden ........ | 4 | 765 |
| Poisoning with corrosive sublimate.. | 1 | 493 |
| Poisoning by arsenic.............. | 3 | 192, 203 |
| Poisoning by opium ............... | 3 | 433 |
| Poisoning from nux vomica........ | 4 | 972 |
| Polypi of the heart .............. | 2 | 564 |
| Pomegranate bark, Mr. Breton on .. | 2 | 872 |
| Poole, Dr. on phrenology.......... | 4 | 851 |
| Popular medicine, Dr. Thomas ..... | 4 | 238 |
| Porter, Dr. liquor morphii citratis .. | 1 | 127 |
| Poultices, evaporating, in gun-shot wounds .................... | 2 | 176 |
| Powell, Dr. on the intestinal canal.. | 1 | 386 |
| Powell, Mr. on injury of the jaw.... | 4 | 766 |
| Power, Dr. on menstruation, abortion, &c.... ............... | 2 | 409 |
| Pregnancy, simulated ............ | 3 | 652 |
| Premature births, Mr. Barlow on.... | 3 | 320 |
| Pretty, Mr. on petechiæ hæmorrhagicæ ................... | 4 | 206 |
| Price, Mr. case of sudden death .... | 2 | 870 |
| Price, Dr. on leeches ............ | 4 | 697 |
| Prichard, Dr. on the nervous system, | 3 | 122 |
| Prichard, Dr. on mania ........... | 3 | 277 |
| Prichard, Dr. on paralysis ....... | 4 | 926 |
| Prince Ruperts, port of .......... | 2 | 591 |
| Pring, Mr. on organic life ......... | 1 | 400 |
| Pring, Dr. on general pathology.... | 4 | 241 |
| Prolapsus ani, treatment of........ | 4 | 191 |
| Prostate gland, remarkable enlargement of ................ | 3 | 72 |
| Prostatic scrofula ................ | 3 | 173 |
| Protracted labours, remarks on .... | 2 | 48 |
| Prout, Dr. on gravel, calculus, &c... | 2 | 98 |
| Prurigo formicans, M. Alibert on ... | 3 | 779 |
| Prussic acid, Dr. Elliotson on ...... | 1 | 639 |
| Prussic acid, Dr. Granville on...... | 1 | 686, 787 |
| Prussic acid .................... | 4 | 940 |
| Psoriasis preputialis, Mr. Evans on.. | 1 | 31 |
| Pterygium....................... | 2 | 374, 641 |
| Puerperal convulsions, Dr. Dewees on | 1 | 128 |
| Puerperal disease, Dr. M. Hall on .. | 1 | 195 |
| Puerperal insanity, Dr. Gooch on .. | 1 | 615 |
| Puerperal mania, Esquirol on ...... | 3 | 269 |
| Puerperal fever, Mr. Moir on ...... | 3 | 648 |
| Puerperal fever, Dr. Payne ........ | 3 | 678 |
| Puerperal fever, Dr. Douglas on .... | 4 | 83 |
| Puerperal convulsions ............ | 4 | 963 |
| Pulmonary disease, obscure case of, | 3 | 185 |
| Pulsations in epigastrio, what? .... | 3 | 22 |
| Pupils, artificial, Dr. Weller on .... | 2 | 449 |
| Purpura hæmorrhagica............ | 3 | 679 |
| Purpura hæmorrhagica............ | 4 | 941 |
| Purulent discharges, Mr. Clark on .. | 3 | 77 |
| Putrefaction, corrector of.......... | 4 | 939 |
| Pyramus frigate, fever in.......... | 4 | 995 |
| **Q** | | |
| Quackery, on the suppression of.... | 2 | 734 |
| Quarantine, Mr. Tully's remarks on.. | 2 | 583 |
| **R** | | |
| Rabbits, Dr. Philip's experiments on, | 2 | 310 |
| Ramsbotham, Dr. on midwifery .... | 2 | 141 |
| Rape ........................... | 4 | 606 |
| Receuil de memoires de chirurgie .. | 3 | 523 |
| Recto-vesical operation ........... | 2 | 337 |
| Rectum, stricture of, Mr. White on, | 1 | 208 |
| Rectum bougies, Mr. Coley on ..... | 1 | 218 |
| Rectum, diseases of, Mr. Howship on, | 1 | 222 |
| Rectum, diseases of, Mr. C. Bell on, | 1 | 264 |
| Rectum, relaxed, Mr. Chevalier on.. | 1 | 465 |
| Rectum, carcinoma of ... ......... | 3 | 84 |
| Rectum, Mr. White on............. | 3 | 326 |
| Rectum, stricture of............... | 3 | 587 |
| Reeder, Dr. on diseases of the heart, | 2 | 401 |
| Reeder, Dr. letter to the reviewers.. | 2 | 686 |
| Reid, Dr. on fever................. | 2 | 327 |
| Remittent, or yellow fever ........ | 3 | 109 |
| Repletion, fatal species of fever from, | 2 | 247 |
| Respiration, remarks of Dr. Ure on.. | 2 | 325 |
| Respiratory nerves, Mr. C. Bell on.. | 2 | 809 |
| Respiratory function, diseases of.... | 3 | 806 |
| Retention of urine .............. | 3 | 773 |
| Retention of urine................. | 4 | 959 |
| Rheumatic metastasis ............ | 4 | 215 |
| Rheumatism, chronic ............. | 3 | 406 |
| Rheumatism, on the treatment of .. | 4 | 933 |
| Rickets, Dr. Weatherhead on ...... | 1 | 153 |
| Rickets, moxa in ................ | 3 | 529 |
| Ringworm of the scalp, works on .. | 2 | 534 |
| Robinson, Dr. on eruptive diseases.. | 2 | 335 |
| Rochoux, M. on apoplexy ......... | 1 | 1 |
| Roots, Dr. on bronchocele ........ | 4 | 801 |
| Roux on abdominal pressure and thoracic percussion .......... | 4 | 569 |
| **S** | | |
| St. Lucie, medical topography of.... | 2 | 590 |
| St. Kitt's, yellow fever there ...... | 4 | 993 |
| Salivation, mode of arresting ...... | 4 | 941 |
| Sandwith, Mr. Humphrey, on fever, | 2 | 203 |
| Sandwith, Mr. Thomas, observations in medicine and surgery.. | 2 | 395 |
| Sangsue, article on, by M. Merat..... | 2 | 348 |
| Sanguineous tumor, Mr. Briggs .... | 4 | 180 |
| Sarcocele, M. Maunoir on.......... | 2 | 682 |
| Scarlet fever, Dr. Macmichael on.... | 3 | 724 |
| Scarpa on scirrhus and cancer ..... | 4 | 172 |
| Scirrhus, Professor Scarpa on...... | 4 | 172 |

| | Vol. | Page |
|---|---|---|
| Sclerotitis | 2 | 376 |
| Scott, Mr. on separation of os uteri, | 2 | 876 |
| Scott, Dr. on dracunculus | 4 | 182 |
| Scott, Dr. on dislocations of the hip-joint | 4 | 342 |
| Scott, Mr. on phrenology | 4 | 854 |
| Scourge of Venus and Mercury | 2 | 597 |
| Scriblomania | 2 | 228 |
| Scrofula, M. Farr on | 1 | 119 |
| Scrofula, Mr. Lloyd on | 3 | 156, 333 |
| Scudamore, Dr. on mineral waters | 1 | 589 |
| Secale cornutum, or ergot of rye | 2 | 442 |
| Semeiology, Dr. Hall on | 4 | 811 |
| Seneca's description of small-pox | 3 | 46 |
| Serres, M. on apoplexy | 1 | 1 |
| Shaw, Mr. on laryngeal disease | 4 | 188 |
| Shaw, Mr. on distortions of the spine | 4 | 882 |
| Shillito, Mr. on ruptured uterus | 4 | 762 |
| Shoulder, dislocations of | 3 | 840 |
| Shoulder-joint, terrible laceration of, | 2 | 240 |
| Simulated diseases | 4 | 596 |
| Sintelaer, Dr. on venereal diseases | 2 | 597 |
| Skull, bony tumor of | 1 | 98 |
| Slow poisons | 4 | 627 |
| Small-pox, modified, Mr. Cross on | 1 | 311 |
| Small-pox, on the antiquity of | 3 | 41 |
| Smerdon, Mr. on arteritis | 3 | 194 |
| Smith, Dr. on emetics | 2 | 282 |
| Smith, Dr. John Gordon, on forensic medicine | 2 | 387 |
| Smith, Dr. Ashby, his edition of Willan | 3 | 41 |
| Solanum tuberosum, Dr. Latham on, | 1 | 384 |
| Spain, fever of, Mr. O'Halloran on | 2 | 817 |
| Spain, degraded state of medicine in, | 2 | 820 |
| Spasmodic diseases, Dr. Moulson on, | 3 | 556 |
| Speer, Dr. on the diseases of Dublin | 4 | 88 |
| Spinal system, Dr. Reid on | 2 | 331 |
| Spinal arachnitis | 2 | 724 |
| Spinal distortion | 3 | 685 |
| Spinal marrow, destruction of | 4 | 701 |
| Spine, scrofula of | 3 | 342 |
| Spine, Mr. Ward on | 3 | 746 |
| Spleen, case of hæmorrhage from | 2 | 334 |
| Sprague's, Mr. formulæ | 4 | 938 |
| Stanley, Mr. case of poisoning | 1 | 638 |
| Staphyloma | 2 | 376 |
| Staphyloma, Dr. Weller on | 2 | 654 |
| Sternalgia, or angina pectoris | 3 | 809 |
| Stethoscope in pregnancy | 3 | 661 |
| Stevenson, Mr. on amaurosis | 2 | 788 |
| Stimulants and contra-stimulants | 2 | 749 |
| Stomach, unsuspected ulcer of | 2 | 446 |
| Stramonium in rheumatism | 3 | 406 |
| Strangulated hernia, curious case of, | 4 | 340 |
| Stricture of the rectum, Mr. White on | 1 | 208 |
| Stricture, spasmodic, of the rectum | 3 | 587 |
| Strictures of the urethra, on | 1 | 528 |
| Strumous ophthalmia | 2 | 371 |
| Strychnine and brucine | 4 | 944 |
| Study of medicine, Dr. Good's | 3 | 574 |
| Subclavian artery, wound of | 2 | 267 |
| Subclavian artery, ligature of | 4 | 954 |
| Subclavian aneurism | 4 | 958 |
| Suicide, tendency to | 3 | 672, 684 |
| Suicide, Esquirol and Falret on | 4 | 1 |
| Suicide, Dr. Paris on | 4 | 625 |
| Surgery, Mr. Barlow on | 3 | 64 |
| Surgical observations, by Mr. T. Sandwith | 2 | 398 |
| Surgical operations, Mr. Averill on, | 4 | 945 |
| Suspended animation, Mr. Whiter on, | 1 | 671 |
| Swan, Mr. on affections of nerves | 2 | 63 |
| Swan, Mr. on the ear | 2 | 873 |
| Swan, Mr. on the action of mercury, | 4 | 277 |
| Swediaur, Dr. on venereal complaints | 2 | 598 |
| Syder, Mr. Myngay, his notes of lectures | 2 | 736 |
| Sympathy, observations on | 2 | 295 |
| Symphysis pubis, division of | 4 | 965 |
| Symptomatology, Sir H. Halford on, | 1 | 634 |
| Symptomatology of indigestion | 2 | 284 |
| Symptomatology, Dr. Hall on | 4 | 811 |
| Syphilis in infants | 1 | 47 |
| Syphilis, Dr. Hennen on | 1 | 283 |
| Syphilis, Dr. Ballingall on | 1 | 565 |
| Syphilis, Dr. Thomson on the origin of | 2 | 616 |

T

| | Vol. | Page |
|---|---|---|
| Tacheron's pathological researches | 4 | 487, 743 |
| Taliacotian operation | 4 | 960 |
| Tar vapour, Dr. Forbes on | 3 | 651 |
| Tar vapour, Sir A. Crichton on | 4 | 102 |
| Tarsus, Mr. Dunn on amputation of, | 2 | 873 |
| Tartrite of antimony, large doses | 3 | 680 |
| Tendo-Achillis, rupture of, treatment | 2 | 740 |
| Testicular scrofula | 3 | 170 |
| Tetanus, successful case of, Mr. Burmester | 2 | 876 |
| Tetanus, case cured | 3 | 181 |
| Tetanus, traumatic | 3 | 423 |
| Tetanus, Mr. Hutchinson on | 4 | 200 |
| Tetanus, Dr. O'Beirne on | 4 | 335 |
| Tetanus, treatment of | 4 | 933 |
| Tetanus, Dr. Coindet on | 4 | 936 |
| Therapeutics, Dr. Chapman on | 4 | 61, 551 |
| Therapeutics | 4 | 935 |
| Thigh, Mr. Amesbury on fractures of, | 3 | 915 |
| Thigh-bone, fractures of | 4 | 707 |
| Thomas, Dr. on the digestive organs, | 2 | 348 |
| Thomas, Dr. on domestic medicine | 4 | 238 |
| Thomson, Dr. J. on mercury | 2 | 598 |
| Thoracic diseases, Drs. Laennec and Forbes on | 2 | 795 |
| Thoracic percussion, on | 4 | 569 |
| Throat, wounds of | 2 | 256 |
| Thyroid gland—a diverticulum? | 3 | 19 |
| Thyroid gland, extirpation of | 4 | 703 |
| Tibia, amputation at the head of | 2 | 243 |
| Tibia, dislocation of | 3 | 833 |

| | Vol. | Page |
|---|---|---|
| Tic-doloureux, Mr. Hutchinson on.. | 1 | 111 |
| Tic-doloureux, Dr. Kerrison on .... | 2 | 63 |
| Tic-doloureux, Mr. Swan on....... | 2 | 68 |
| Tic-doloureux, remarks on ........ | 3 | 30 |
| Tic-doloureux .................. | 3 | 176 |
| Tic-doloureux, Mr. Hutchinson on / Tic-doloureux, Dr. Yeats on ..... / Tic-doloureux, Dr. Palmer on.... | 3 | 495 |
| Tic-doloureux, Dr. Wilson on...... | 3 | 691 |
| Tic-doloureux, iron in ............ | 4 | 700 |
| Tincture of poppy, Dr. Wilson on .. | 2 | 669 |
| Tobacco in the phlegmasiæ ........ | 3 | 414 |
| Todd, Mr. on axillary aneurism..... | 3 | 402 |
| Todd, Mr. on aneurism............ | 4 | 77 |
| Todd, Mr. on diseases of lachrymal gland ........................ | 4 | 344 |
| Tommasini on the new Italian doctrines ..................... | 2 | 745 |
| Tommasini on English medicine.... | 3 | 151 |
| Tongue, ulcerations of ............ | 3 | 543 |
| Topography, medical, of Ceylon .... | 3 | 53 |
| Tourniquets in amputation ........ | 4 | 953 |
| Tracheotomy, case of............. | 1 | 318 |
| Tracheotomy, cases of, Mr. Carmichael ...................... | 2 | 516 |
| Tracheotomy, case of, by Mr. Porter, | 2 | 878 |
| Tracheotomy, curious case of ...... | 4 | 955 |
| Transactions of the New York Medical Society ................ | 2 | 275 |
| Transactions of the Irish College, vol. iii. ...................... | 2 | 327 |
| Transactions of the King's & Queen's College ................... | 2 | 511 |
| Transactions, Medico-Chirugical.... | 3 | 386 |
| Transactions of the Apothecaries.... | 4 | 519 |
| Transactions of the Medico-Chirurgical Society ................ | 4 | 790 |
| Transactions of the Phrenological Society..................... | 4 | 847 |
| Transfusion of blood, Dr. Blundell on | 1 | 117 |
| Travers, Mr. on diseases of the eye.. | 2 | 105, 369 |
| Treatment of indigestion .......... | 2 | 297 |
| Trephine, seldom necessary........ | 2 | 250 |
| Triumph (74), mercurial vapour in.. | 4 | 1010 |
| Tropical climates, Dr. Chisholm on.. | 3 | 100 |
| Tuberculous diseases, Dr. Baron on.. | 3 | 785 |
| Tully, Mr. on plague ............. | 2 | 569 |
| Tumor, immense, removed by Sir A. Cooper .................... | 2 | 880 |
| Tumor, encysted, curious case...... | 4 | 915 |
| Tumor, immense, removed by Mr. Liston ..................... | 4 | 957 |
| Tunbridge Wells, mineral waters of, | 1 | 594 |
| Tympanites intestinalis............ | 3 | 193 |
| **U** | | |
| Ulcer in the stomach, unsuspected.. | 2 | 446 |
| Ulcer, malignant, of the prepuce.... | 3 | 405 |
| Ulceration of the cæcum .......... | 4 | 739 |
| Ulcerations of the genital organs.... | 1 | 28 |
| Urethra, bursting of, Mr. Bell on .. | 1 | 477 |
| Urethra, strictures of ............ | 1 | 528 |
| Urethra, Mr. Arnott on the........ | 1 | 727 |
| Urethra, muscular fibres in? ...... | 2 | 555 |
| Urethra, Mr. Earle on ............ | 2 | 858 |
| Urethra (female) dilatation of...... | 4 | 193 |
| Urethra of the male, Dr. Kerrison on, | 4 | 802 |
| Urinary calculi, table of .......... | 1 | 93 |
| Urinary organs, Mr. C. Bell on the.. | 1 | 264 |
| Urinary organs, Mr. Wilson on .... | 3 | 260 |
| Urinary organs, Mr. Howship on the, | 4 | 369 |
| Urinary secretion, physiology of.... | 4 | 965 |
| Urine, on the physiology and pathology of ................. | 2 | 41 |
| Urine, involuntary discharge of .... | 2 | 788 |
| Urine, suppression and retention of.. | 3 | 265 |
| Urine, curious case of suppression of, | 4 | 372 |
| Urine, suppression of............. | 4 | 959 |
| Urine, retention of............... | 4 | 959 |
| Uterine hæmorrhage, Mr. Williams on | 1 | 155 |
| Uterine hæmorrhage, Dr. Campbell on | 1 | 470 |
| Uterine disease, obscure case of .... | 2 | 445 |
| Uterine inflammation............. | 3 | 78 |
| Uterine epilepsy ................ | 3 | 132 |
| Uterine mania.................. | 3 | 285 |
| Uterine hæmorrhage, Dr. Gooch on.. | 3 | 662 |
| Uterus, extirpation of ............ | 1 | 104 |
| Uterus, rupture of, not fatal ...... | 1 | 108 |
| Uterus, Dr. Douglas on the hourglass contraction of the ....... | 1 | 631 |
| Uterus, remarkable contraction of .. | 3 | 426 |
| Uterus, retroversion of........... | 3 | 673 |
| Uterus, cancer of, extirpation ...... | 4 | 463 |
| Uterus, rupture of, Mr. Shillito on.. | 4 | 762 |
| Uvula, elongated................ | 3 | 681 |
| Uvula, elongation of............. | 4 | 956 |
| **V** | | |
| Vacca, Professor, on recto-vesical operation ................... | 2 | 337 |
| Vaccination, Mr. Cross on ........ | 1 | 302 |
| Vaccination, Sir G. Blane on ...... | 1 | 702 |
| Vaccination, Dr. Jenner on ........ | 1 | 702 |
| Vaccination, Dr. Gregory on ...... | 4 | 806 |
| Vaccine and measles at once ...... | 4 | 205 |
| Vagina, ruptured, case of.......... | 2 | 530 |
| Vaginal inflammation ............ | 3 | 79 |
| Varicella ...................... | 1 | 309 |
| Varicose veins ................. | 4 | 954 |
| Variola and varicella, identity of.... | 3 | 198 |
| Variola and varicella............. | 4 | 214 |
| Variolous epidemic in Norwich .... | 1 | 300 |
| Variolous epidemic, Dr. Forbes on.. | 3 | 646 |
| Venereal disease in the fœtus ...... | 4 | 839 |
| Venerola, varieties of, description of the...................... | 1 | 34 |
| Ventriloquism ................ .. | 2 | 219 |
| Verdigrease, poisoning by ........ | 1 | 158 |
| Verminous diseases, Prof. Brera on, | 1 | 549 |
| Vessels, erectile powers of ......... | 3 | 24 |
| Vetch, Dr. on the diseases of the eye, | 1 | 652 |
| Veterinary art, lectures on ........ | 4 | 583 |
| Villers, Dr. on tic-doloureux ...... | 2 | 63 |

| | Vol. | Page |
|---|---|---|
| Vincent, Mr. operation on the neck, | 4 | 207 |
| Viscera, morbid anatomy of........ | 2 | 553 |
| Voltaic battery .................. | 3 | 206 |
| Vomiting, effects of, in apoplexy.... | 3 | 550 |

W

| | Vol. | Page |
|---|---|---|
| Wallace, Mr. on sulphureous fumigation.................... | 2 | 507 |
| Wallace, Mr. on chlorine.... ..... | 3 | 510 |
| Ward, Mr. on spinal distortions .... | 3 | 746 |
| Wardrop, Mr. on inordinate irritability ..................... | 1 | 485 |
| Wardrop, Mr. on wounded nerves .. | 4 | 450 |
| Water-stroke, (wasserschlag), Dr. Golis on.................... | 2 | 466 |
| Waters, mineral, Dr. Scudamore on<br>Waters, mineral, Dr. Mackenzie on | 1 | 589 |
| Watery discharge from the uterus .. | 2 | 777 |
| Weatherhead, Dr. on rickets ...... | 1 | 153 |
| Webb, Dr. on the Egyptian plague .. | 1 | 391 |
| Webster, Dr. on hooping-cough .... | 4 | 449 |
| Weller, Dr. on diseases of the eye .. | 2 | 626 |
| White, Mr. on stricture of the rectum | 1 | 208 |
| White, Mr. on the rectum ........ | 3 | 326 |
| Whiter, Mr. on suspended animation, | 1 | 671 |
| Wilbank, Mr. on sloughing phagedena | 2 | 875 |
| Wilkinson, Mr. on cutaneous diseases | 4 | 132 |
| Willan, Dr. his miscellaneous works, | 3 | 41 |
| Williams on uterine hæmorrhage ... | 1 | 155 |
| Wilson, Mr. lectures on osteology .. | 1 | 411 |
| Wilson, Mr. on the urinary organs.. | 3 | 260 |
| Wilson, Mr. account of his death .. | 3 | 267 |
| Windsor, Mr. case of extirpation of the uterus.......... .... | 1 | 104 |
| Wishart, Mr. case of ligature of the subclavian ................. | 4 | 958 |
| Worms, intestinal, description of.... | 1 | 549 |
| Wounds of the chest, Larrey on .... | 1 | 446 |
| Wounds of the intestines, Larrey on, | 1 | 473 |
| Wounds, gun-shot, Messrs. Guthrie, Hennen, Laurent, and Percy on ......................... | 2 | 165 |
| Wounds of head, Baron Larrey on .. | 2 | 249 |
| Wounds in dissecting, treatment of.. | 2 | 552 |
| Wounds from dissection, treatment of | 2 | 739 |
| Wounds, poisoned ............... | 4 | 698 |

Y

| | Vol. | Page |
|---|---|---|
| Yeats, Dr. on strangulated hernia .. | 1 | 373 |
| Yeats, Dr. on the duodenum ...... | 1 | 623 |
| Yeats, Dr. G. D. on neuralgia ...... | 3 | 495 |
| Yellow fever, Dr. Jackson on ...... | 2 | 485 |
| Yellow fever, Dr. Ferguson on ..... | 2 | 594 |
| Yellow fever, Mr. O'Halloran on.... | 2 | 817 |
| Yellow fever, Dr. Chisholm on ..... | 3 | 109 |
| Yellow fever, Mr. Coventry on .... | 3 | 184 |
| Yellow fever, Sir Gilbert Blane on .. | 3 | 739 |
| Yellow fever, Dr. Musgrave on .... | 4 | 979 |
| Yellow fever, Dr. Hartle on........ | 4 | 993 |

Printed by G. Hayden, Little College Street, Westminster.

